# Relative band power in assessing temporary neurological dysfunction post- type A aortic dissection surgery: a prospective study

**DOI:** 10.1038/s41598-024-58557-y

**Published:** 2024-04-03

**Authors:** Ya-peng Wang, Li Li, Hua Jin, Yang Chen, Yi Jiang, Wen-xue Liu, Yun-xing Xue, Li Huang, Dong-jin Wang

**Affiliations:** 1 Department of Cardio-Thoracic Surgery, Nanjing Drum Tower Hospital, Chinese Academy of Medical Sciences & Peking Union Medical College, Beijing, China; 2 Department of Cardio-Thoracic Surgery, Nanjing Drum Tower Hospital, The Afliated Hospital of Nanjing University Medical School, Nanjing, China; 3 Department of Critical Care Medicine, Xiangya Hospital of Central South University, Changsha, 410008 Hunan People’s Republic of China

**Keywords:** Aortic Dissection, Temporary neurological dysfunction, Delirium, Delayed recovery, Relative band power, Cardiopulmonary bypass, Neuroscience, Cardiovascular diseases, Neurological disorders

## Abstract

Temporary neurological dysfunction (TND), a common complication following surgical repair of Type A Aortic Dissection (TAAD), is closely associated with increased mortality and long-term cognitive impairment. Currently, effective treatment options for TND remain elusive. Therefore, we sought to investigate the potential of postoperative relative band power (RBP) in predicting the occurrence of postoperative TND, with the aim of identifying high-risk patients prior to the onset of TND. We conducted a prospective observational study between February and December 2022, involving 165 patients who underwent surgical repair for TAAD at our institution. Bedside Quantitative electroencephalography (QEEG) was utilized to monitor the post-operative brain electrical activity of each participant, recording changes in RBP (RBP Delta, RBP Theta, RBP Beta and RBP Alpha), and analyzing their correlation with TND. Univariate and multivariate analyses were employed to identify independent risk factors for TND. Subsequently, line graphs were generated to estimate the incidence of TND. The primary outcome of interest was the development of TND, while secondary outcomes included intensive care unit (ICU) admission and length of hospital stay. A total of 165 patients were included in the study, among whom 68 (41.2%) experienced TND. To further investigate the independent risk factors for postoperative TND, we conducted both univariate and multivariate logistic regression analyses on all variables. In the univariate regression analysis, we identified age (Odds Ratio [OR], 1.025; 95% CI, 1.002–1.049), age ≥ 60 years (OR, 2.588; 95% CI, 1.250–5.475), hemopericardium (OR, 2.767; 95% CI, 1.150–7.009), cardiopulmonary bypass (CPB) (OR, 1.007; 95% CI, 1.001–1.014), RBP Delta (OR, 1.047; 95% CI, 1.020–1.077), RBP Alpha (OR, 0.853; 95% CI, 0.794–0.907), and Beta (OR, 0.755; 95% CI, 0.649–0.855) as independent risk factors for postoperative TND. Further multivariate regression analyses, we discovered that CPB time ≥ 180 min (OR, 1.021; 95% CI, 1.011–1.032), RBP Delta (OR, 1.168; 95% CI, 1.105–1.245), and RBP Theta (OR, 1.227; 95% CI, 1.135–1.342) emerged as independent risk factors. TND patients had significantly longer ICU stays (p < 0.001), and hospital stays (p = 0.002). We obtained the simplest predictive model for TND, consisting of three variables (CPB time ≥ 180 min, RBP Delta, RBP Theta, upon which we constructed column charts. The areas under the receiver operating characteristic (AUROC) were 0.821 (0.755, 0.887). Our study demonstrates that postoperative RBP monitoring can detect changes in brain function in patients with TAAD during the perioperative period, providing clinicians with an effective predictive method that can help improve postoperative TND in TAAD patients. These findings have important implications for improving clinical care in this population.

Trial registration ChiCTR2200055980. Registered 30th Jan. 2022. This trial was registered before the first participant was enrolled.

## Introduction

Type A Aortic Dissection (TAAD) poses a formidable challenge for cardiac surgeons, even with the considerable progress in medical technology achieved over the past five decades. Among the various brain protection techniques employed during surgical interventions for TAAD, hypothermic circulatory arrest (HCA) and the selective cerebral perfusion strategy have become significant measures for brain protection have become as the most commonly method^1,2^. The utilization of HCA has substantially improved the efficacy of surgical interventions. Nevertheless, postoperative cerebral complications remain a significant concern for medical professionals^3^. Transient neurological dysfunction (TND) , such as postoperative delirium, can afflict a substantial number of patients undergoing surgery, with reported incidence rates as high as 40%^4–6^. This condition can significantly prolong the duration of ICU stays and escalate overall hospitalization costs^5^. Recent research has also indicated that postoperative delirium may precipitate enduring cognitive impairment, particularly among elderly populations^7–9^.

Despite extensive research on pre- and intraoperative risk factors in patients with TAAD^3,10–12^, there is limited investigation into brain function changes during the perioperative period. Although intraoperative cerebral oxygen monitoring has become standard practice for TAAD, its diagnostic accuracy for postoperative neurological dysfunction remains inadequate^13^. Conversely, electroencephalography (EEG) boasts high sensitivity for cortical ischemia and brain dysfunction, enabling early detection of localized or generalized brain impairment^14^. However, the interpretation of EEG signals poses significant challenges, making it challenging to incorporate into the realm of cardiac surgery^14^.

Quantitative electroencephalography (QEEG) is a valuable tool for non-neurologists to diagnose and treat brain function disorders due to its simplicity of use and capacity to directly measure neuronal activity. An essential parameter of QEEG is relative band power (RBP), which reflects the proportional distribution of delta, theta, beta and alpha waves. In perioperative patients, increases in delta and theta waves, coupled with decreases in alpha waves, are closely correlated with transient neurological dysfunction (TND) and cognitive impairment^15,16^.

The predictive utility of relative band power (RBP) in gauging brain function prognosis in patients with TAAD is yet to be determined. To bridge this knowledge gap, this study advocates for the adoption of QEEG to track brain function in TAAD patients during the perioperative period. These findings could have profound implications for diagnosing, treating, and predicting the prognosis of brain function disorders in TAAD patients.

## Methods

### Study design, setting and participants

**Figure 1:**
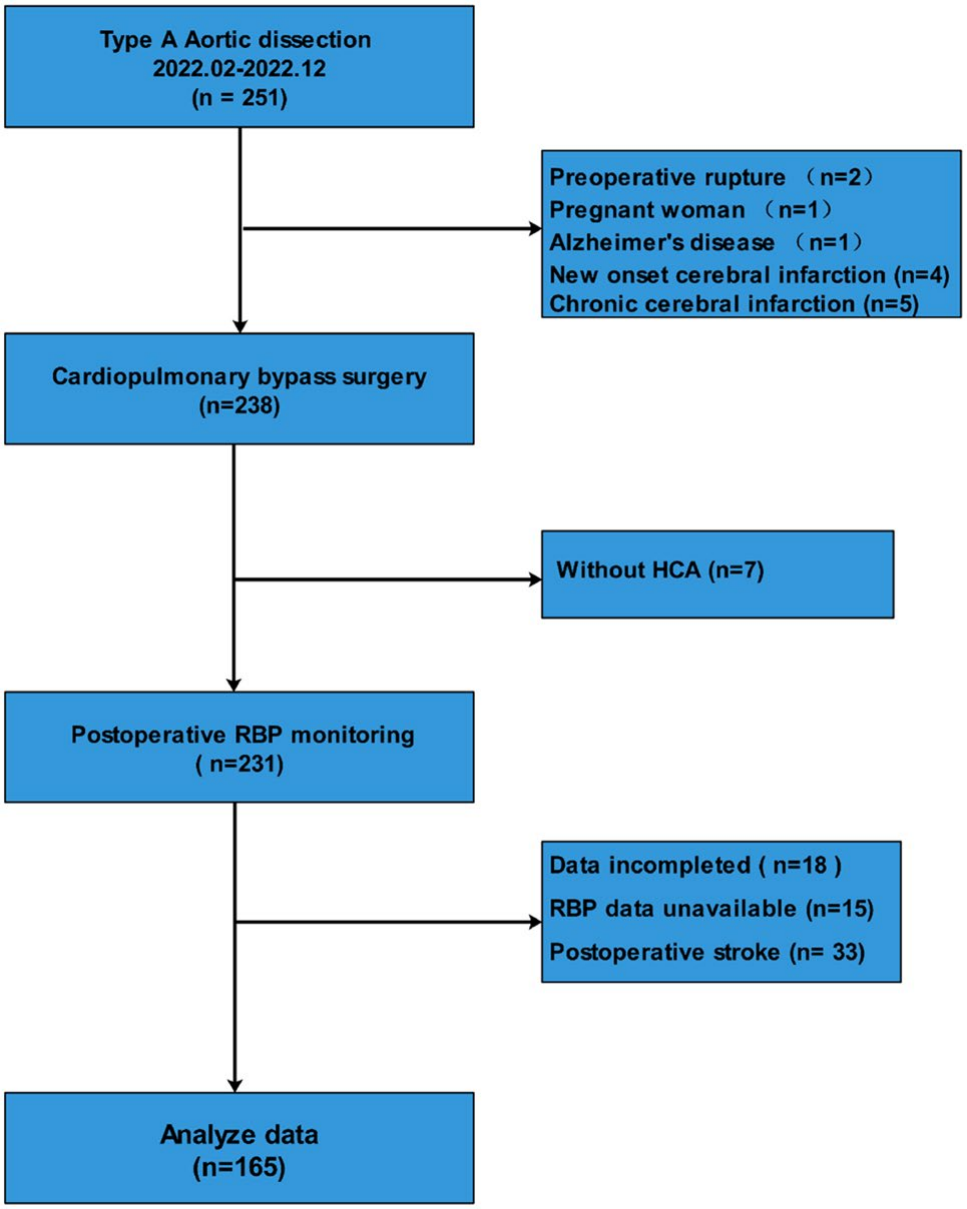
Consolidated standards of reporting trials diagram demonstrating selection of patients undergoing TAAD surgery repair. *RBP* relative band power.

A prospective observational study was conducted at the Department of Cardio-Thoracic Surgery of Nanjing Drum Tower Hospital of the Affiliated Hospital of Nanjing University Medical School between February 2022 and December 2022. Institutional review board approval was obtained from The Nanjing Drum Tower Hospital of the Affiliated Hospital of Nanjing University Medical School prior to the study, which was registered on the Chinese Clinical Trial Registry (ChiCTR2200055980). All patients provided written informed consent after receiving information sheets and potential risk disclosures. The study included patients over the age of 18 who underwent open surgery under general anesthesia for TAAD. The exclusion criteria are as follows: (1) Patients diagnosed with malignancies that significantly impact life expectancy are excluded; (2) Patients who have experienced a cerebral infarction, been in a state of pregnancy, or have a history of psychiatric illness within the past six months are excluded; (3) Patients presenting with a new cerebral infarction caused by aortic dissection; (4) Patients with concurrent serious diseases unrelated to aortic dissection, which are expected to impact life expectancy, are also excluded. (5) Excluding patients with indeterminate postoperative wake-up times. (6) Excluding patients with postoperative cerebral infarction was a strategic decision, informed by our objective to investigate the effects of RBP on TND among TAAD patients devoid of preoperative cerebral symptoms or postoperative strokes. This methodological choice was intended to diminish confounding variables, facilitating a concentrated examination of RBP's potential protective or predictive capacities within a more homogeneous patient cohort. Moreover, this exclusion criterion aimed to refine the study's focus, sidestepping the intricate and diverse neurological impacts associated with postoperative cerebral infarctions, which could potentially cloud the insights pertaining to TND. The study enrolled a total of 165 patients (Fig. 1), and measures were taken to maintain the validity and robustness of the research findings.

### Surgical procedures

All patients underwent venous, inhalational anesthesia, and were subjected to endotracheal intubation, arterial puncture for blood pressure monitoring in both the upper and lower extremities, and the placement of an esophageal ultrasound probe. The right axillary artery and right femoral artery were exposed, followed by a median sternotomy to reveal the supra-arch vessels. Subsequent to systemic heparinization, CPB was initiated through both the right axillary and femoral arteries, facilitating venous drainage into the right atrium. Retrograde myocardial perfusion was routinely administered via the coronary sinus. Upon reducing the bladder temperature to 24–28℃, circulatory arrest was induced, employing selective cerebral perfusion (SCP) from the right axillary artery for brain perfusion. Retrograde Cerebral Perfusion (RCP) was administered through the Superior Vena Cava during CPB, utilizing either a Y-shaped arterial connector or direct SVC cannulation. The perfusion pressure was maintained between 30–40 mmHg to ensure optimal perfusion efficacy. Throughout the circulatory arrest period, cerebral oxygen saturation was continuously monitored, aiming to keep cerebral oxygenation levels within ± 20% of the baseline values. Distal repair techniques were tailored to the specific clinical scenario. In general, patients presenting with arch dilation (≥ 45 mm), an intimal tear in the arch, or structural arch damage underwent total arch replacement utilizing a quadrifurcated graft. The frozen elephant trunk technique was also employed in conjunction with total arch replacement. Alternatively, partial arch replacement or antegrade stent grafting in the aortic arch (a technique pioneered by our center) was implemented^17–19^. Following the completion of anastomosis, cardiac temperature recovery was initiated. During the rewarming phase, aortic root repair was performed. This involved the excision of all thrombi within the aortic root dissection, the placement of a Dacron patch shaped to fit between the adventitia and intima of the aortic root as a new media, insertion of a Dacron felt inside the intima, and continuous suturing of the newly established four-layer aortic root.

### RBP recording

**Figure 2:**
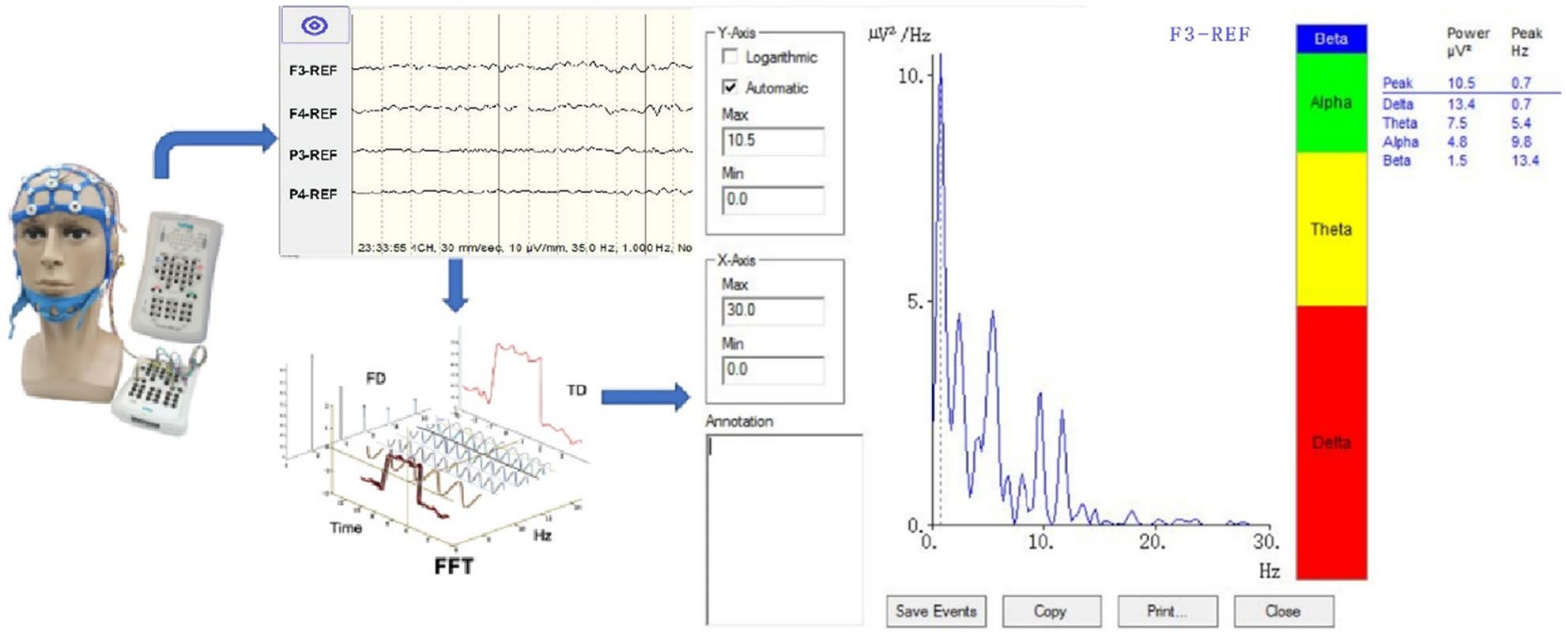
QEEG collects the raw EEG signal from the scalp, analyzes it by fast Fourier transform (FFT), and displays the spectrum and power spectrum of the EEG sequence signal in the form of a trend spectrum. *FD* frequency domain, *TD* time domain.

Bedside QEEG using a brain function instrument (Nicolet Monitor, NicoletOne 5.9.4, Natus Neurology Incorporated) was employed to monitor brain activity in each participant 2 h post-surgery. Dual-channel recordings were obtained from the scalp positions C3, P3, C4, and P4, defined by the international 10–20 EEG system^20^. Computer-generated algorithms implemented in the data recording device were used to filter and compress the raw data from each hemisphere. RBP used different colors to represent the occupancies of delta, theta, beta and alpha waves in the trend graph (see Fig. 2). Stringent criteria were applied to assess the quality of the recorded data, including electrode impedance below 10 kΩ, checking for motion artifacts and ECG interference in the raw data, and ruling out potential interference from diathermy or other electrical equipment.

### Definition of end-points

TND is a composite measure defined as the combination of postoperative delirium (POD) and delayed recovery. In surgical survivors without permanent neurological dysfunction, POD is diagnosed using the Confusion Assessment Method for the ICU (CAM-ICU)^21^ and assessed twice daily for seven consecutive days. Delayed recovery refers to delayed emergence from anesthesia after surgery, and its definition remains controversial in clinical practice. In our CICU, in accordance with the prevailing consensus, the therapeutic time window for acute ischemic stroke thrombolysis is currently confined to a 6 h interval. To expedite the detection of early stroke manifestations, our research center designates patients who remain unresponsive within 6 h post-surgery as experiencing "delayed recovery." We utilize brain computed tomography angiography and perfusion examinations to promptly evaluate their condition, enabling the prompt formulation of an optimal treatment strategy. By adopting this methodology, our overarching aim is to capitalize on the prime therapeutic opportunity within the designated time window, thereby enhancing treatment outcomes for afflicted individuals.

### Statistical analysis

The sample size calculation was conducted utilizing the Power Analysis & Sample Size software (version 15.0, NCSS statistical software, Kaysville, UT, USA)^22–24^. A thorough review of the literature indicated a prevalence range of POD following TAAD surgery between 32.5% and 52.0%^25,26^. Literature findings have suggested an EEG-predicted occurrence rate of delirium at 60%^27^. For this study, a conservative estimate of 45% prevalence was adopted to ensure more precise estimations^28^. To attain a statistical power of 90% at a significance level of 0.05, as determined by Student's t-test, an initial total sample size of 115 patients was calculated. The sample size was subsequently increased by 20% to accommodate potential attrition and other variables, yielding a final sample size of 144 patients.

In this study, we assessed the normality of continuous variables using the Kolmogorov–Smirnov test. Continuous variables that followed a normal distribution were reported as mean ± standard deviation and analyzed using Student's t-test. Non-normally distributed continuous variables were reported as median with interquartile ranges (Q1–Q3) and analyzed using the Mann–Whitney U-test. Categorical variables were presented as frequencies and percentages (n, %) and analyzed using either the chi-squared test or Fisher's exact test. All statistical analyses were two-tailed, and statistical significance was defined as a P-value less than 0.05. Furthermore, we employed single-variable binary logistic regression analysis to evaluate the relationship between different variables and the outcome, calculating the odds ratios (OR) and 95% confidence intervals (CI).

### Model development

Subsequently, variables with a P-value less than 0.05 were included in a stepwise (backward: conditional) multivariate logistic regression analysis model to establish a predictive model. Additionally, we constructed a nomogram using variables with a P-value less than 0.05 in the multivariate analysis to aid in clinical decision-making.

### Model performance and internal validation

Subsequently, we evaluated the performance of the nomogram through internal validation using the bootstrap method with 1000 resamples, assessing both discrimination and calibration. Discrimination was measured by the C-statistic, which was equivalent to the area under the receiver operating characteristic (ROC)^29^. Calibration was evaluated by plotting calibration curves and calculating the Brier score using the equation (Y-p)^2^, where Y represented the actual observed outcome and *P* represented the predicted probability from the nomogram^30^. In addition to developing quantitative nomograms for predicting the probability of postoperative neurological complications in aortic surgery, a decision curve analysis (DCA) was performed to assess the clinical applicability of the nomograms^31^. The DCA quantified the clinical net benefit of the predictive model at different threshold probabilities, providing valuable insights into the practical use of the model in clinical decision-making. The statistical analysis was performed using SPSS 25.0 and R 4.1.1, and *P* < 0.05 was considered statistically significant.

### Ethics approval and consent to participate

The Institutional Review Board of Nanjing Drum Tower Hospital approved the study and protocol (approval number: 2022-034-01). The trial was conducted in accordance with the ethical principles of the “Declaration of Helsinki,” the “Ethical Review Measures for Biomedical Research Involving Humans” of the National Health Commission of China, and other relevant national laws and regulations. Informed consent by the study participant or a legally authorized representative was given prior to inclusion in the study.

## Results

### Patient characteristics and neurological outcomes

During the study period from February 1, 2022, to January 1, 2023, a total of 165 patients were included in our analysis, as depicted in Fig. 1. Preoperative consciousness disorders were observed in 15 cases (9.1%). 33 patients with postoperative strokes were excluded. The overall incidence rate of TND was 41.2%. In Table 1, our analysis revealed several factors that significantly increased the likelihood of TND development, including: age (p = 0.036), Hemopericardium (p = 0.039), concomitant CABG (p = 0.011), CPB time (p = 0.046), P/F (p = 0.015). TND patients had significantly longer ICU stays (p < 0.001), and hospital stays (p = 0.002). Additionally, the TND group exhibited significant differences in Post-RBP Delta, Alpha, and Beta values compared to the non-TND group.

**Table 1:** Basic Characteristics in TND and Non-TND groups.

Characteristic	TND (N = 68)	Non-TND (N = 97)	*P-Value*
Sex Male (N, %)	53 (77.9)	77 (79.4)	0.977
BMI (kg/m^2^) ^b^	25.31 (23.61–27.76)	26.10 (23.50–29.40)	0.503
Age (years)^b^	53.00 (41.75–66.50)	49.00 (41.00–58.00)	**0.07**
Age ≥ 60 years (N, %)	23 (33.8)	16 (16.5)	**0.017**
Hypertension (N, %)	51 (75.0)	65 (67.0)	0.351
CAD (N, %)	2 (2.9)	2 (2.1)	1.000
Hepatitis (N, %)	3 (4.4)	3 (3.1)	0.691
Allergies (N, %)	3 (4.4)	9 (9.3)	0.363
Nephropathy (N, %)	4 (5.9)	3 (3.1)	0.448
MI (N, %)	1 (1.5)	0 (0.0)	0.412
AF (N, %)	1 (1.5)	0 (0.0)	0.412
Immune diseases (N, %)	0 (0.0)	5 (5.2)	0.078
Marfan syndrome (N, %)	1 (1.5)	3 (3.1)	0.644
Malignancy (N, %)	1 (1.5)	1 (1.0)	1.000
Smoking (N, %)	15 (22.1)	18 (18.6)	0.693
Alcohol (N, %)	16 (21.6)	21 (18.6)	0.610
Consciousness disorders (N, %)	9 (13.2)	6(6.2)	0.2
Reoperation (N, %)	0 (0.0)	2 (2.1)	0.512
Hemopericardium (N, %)	15 (22.1)	9 (9.3)	**0.039**
Urgency of operation (N, %)			0.802
Urgent	63 (92.6)	92 (74.8)	
Emergent	5 (7.4)	5 (5.2)	
Intraoperative data			
Mesenteric ischemia (N, %)	2 (2.9)	0 (0.0)	0.168
AVR (N, %)	12(17.6)	24(24.7)	0.371
CABG (N, %)	5 (7.4)	0(0.0)	**0.011**
ECMO (N, %)	1(1.5)	0 (0.0)	0.412
Cerebral perfusion (N, %)			0.361
u-ASCP	54 (79.4)	76 (78.4)	
RCP	4 (5.9)	3 (3.1)	
Bi-ASCP	9 (13.2)	18 (18.6)	
DHCA	1 (1.5)	0 (0.0)	
Lowest temperature (N, %)			1.000
20.0–24.0℃	52 (76.5)	74 (76.3)	
24.1–28.0℃	16 (23.5)	23 (23.7)	
Aortic procedure (N, %)			0.263
Hemi-arch	17 (25.0)	14 (14.4)	
Fenestrated arch stent	15 (22.1)	18 (18.6)	
Island-total arch replacement	16 (23.5)	31 (32.0)	
Total arch replacement	20 (29.4)	34 (35.1)	
Operative time (min)^b^	382.50 (331.25–420.00)	380.00 (330.00–402.50)	0.381
CPB time (min)^b^	188.00 (164.25–222.25)	173.00 (142.50–213.00)	**0.046**
CPB ≥ 180 min (N, %)	42 (61.8)	45 (46.4)	0.074
Aortic cross-clamp time (min)^b^	135.00 (106.50–164.75)	127.00 (108.00–160.50)	0.331
HCA (min)^b^	28.00 (23.00–34.75)	28.00 (23.00–33.50)	0.528
Postoperative data			
P/F	86.44 (66.24–169.61)	125.33 (83.71–228.89)	**0.015**
Post-RBP data			
Delta (%)^a^	71.80 ± 12.00	65.03 ± 12.52	** < 0.001**
Theta (%)^b^	14.62 (9.21–19.67)	12.62 (9.20–18.16)	**0.185**
Alpha (%)^b^	7.17 (4.41–10.89)	12.76 (9.93–17.86)	** < 0.001**
Beta (%)^b^	2.97 (1.86–4.32)	4.88 (3.15–9.02)	** < 0.001**
CCU day	6.00 (4.00–9.00)	4.00 (3.00–5.00)	** < 0.001**
Hospital stays	19.00 (15.00–24.00)	15.00 (13.00–20.00)	**0.002**

^a^is expressed as mean ± standard deviation, ^b^Values are expressed as interquartile spacing (median (¼ -¾ digits)), *AVR* Aortic Valve Replacement, *BMI* Body mass index, *CAD* coronary artery disease, *MI* myocardial infarction, *AF* atrial fibrillation, *CABG* coronary artery bypass grafting, *ECMO* extracorporeal membrane oxygenation, *u-ASCP* unilateral antegrade selective cerebral perfusion, *Bi-ASCP* bilateral antegrade selective cerebral perfusion, *RCP* retrograde cerebral perfusion, *DHCA* deep hypothermic circulatory arrest, *MHCA* moderate hypothermic circulatory arrest, *CPB* cardiopulmonary bypass, *HCA* hypothermic circulatory arrest, *P/F* PaO2/FiO2 ratio.

Significant values are bold.

To delve deeper into the independent risk factors associated with postoperative TND, comprehensive univariate and multivariate logistic regression analyses were performed on all pertinent variables. The univariate analysis delineated several variables as independent predictors of postoperative TND, including age (OR 1.025, 95% Confidence Interval [CI] 1.002–1.049), age ≥ 60 years (OR 2.588, 95% CI 1.250–5.475), presence of hemopericardium (OR 2.767, 95% CI 1.150–7.009), CPB time (OR 1.007, 95% CI 1.001–1.014), RBP Delta (OR 1.047, 95% CI 1.020–1.077), RBP Alpha (OR 0.853, 95% CI 0.794–0.907), and RBP Beta (HR 0.755, 95% CI 0.649–0.855) in Table 2. Advancing to multivariate regression analysis, it was unveiled that an extended CPB time ≥ 180 min (OR 1.021, 95% CI 1.011–1.032), RBP Delta (OR 1.168, 95% CI 1.105–1.245), and RBP Theta (OR 1.227, 95% CI 1.135–1.342) stood out as significant independent risk factors for the development of TND postoperatively in Table 3.

**Table 2:** Univariate regression analysis identified independent risk factors for TND.

Variables	*B*	OR	95% CI
Age (years)	0.025	1.025	1.002–1.049
Age ≥ 60 (years)	0.951	2.587	1.250–5.475
Hemopericardium	1.018	2.767	1.150–7.009
CPB (min)	0.007	1.007	1.001–1.014
RBP Delta	0.046	1.047	1.020–1.077
RBP Alpha	− 0.159	0.853	0.794–0.907
RBP Beta	− 0.281	0.755	0.649–0.855

*Β* regression coefficient, *OR* Odds ratio, 95% *CI*: 95% confidence interval. *CPB* cardiopulmonary bypass.

**Table 3:** Multivariable logistic regression analysis of independent risk factors for TND.

Variables	*Β*	*OR*	95% *CI*
CPB ≥ 180 (min)	0.021	1.021	1.011–1.032
RBP delta	0.155	1.168	1.105–1.245
RBP theta	0.205	1.227	1.135–1.342

*Β* regression coefficient; *OR* Odds ratio; 95% *CI*: 95% confidence interval. *CPB* cardiopulmonary bypass.

### Development and Validation of the model for TND

**Figure 3:**
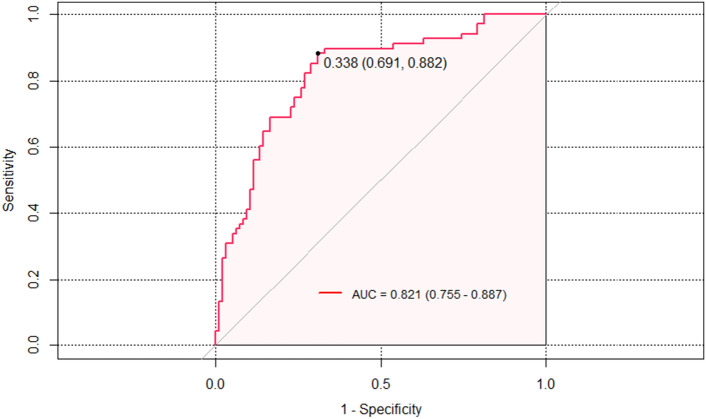
AUROC curve analysis of the Model for TND Post-RBP Delta and Theta; AUROC = 0.800 which is equal to a c-statistic.

**Figure 4:**
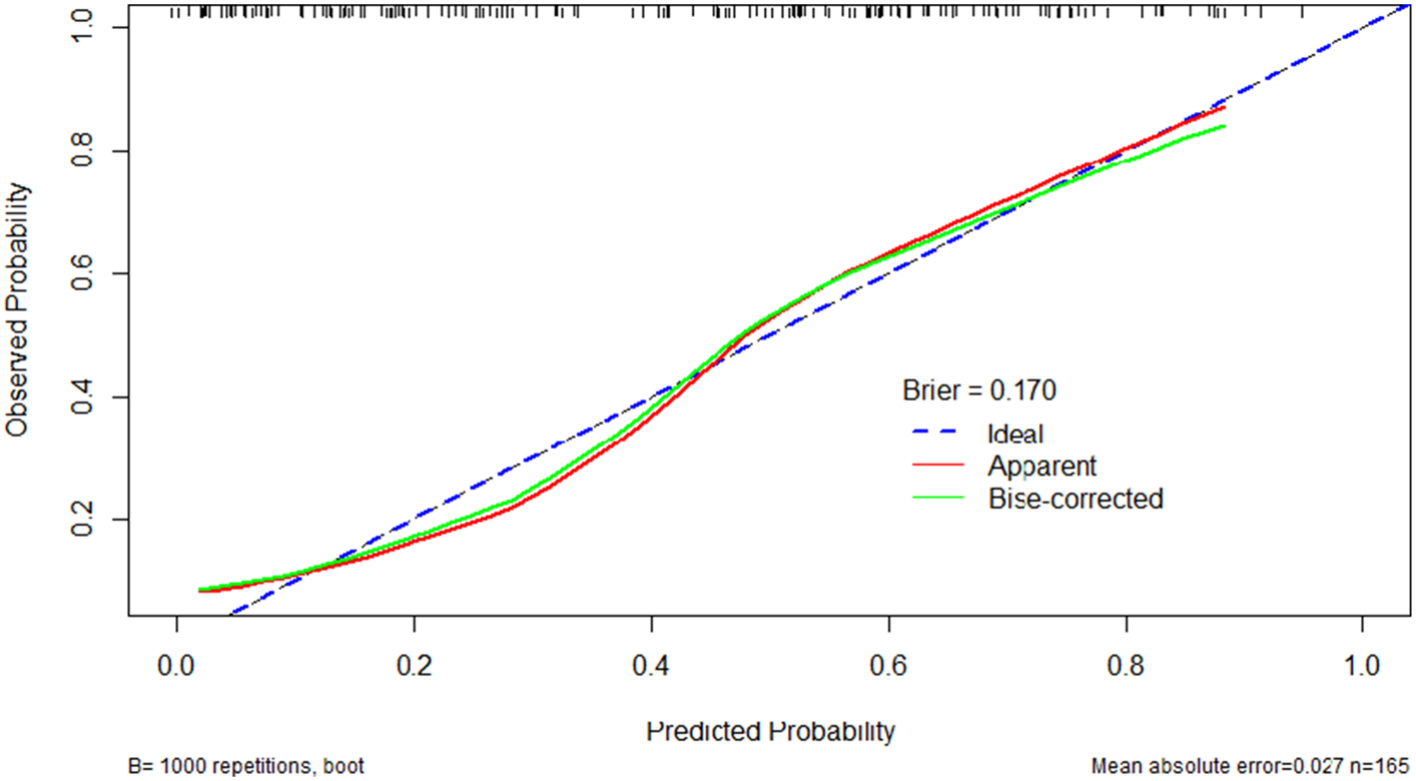
Internal calibration curves for Model. The Model perfectly accurate model would produce a graph where the observed and predicted probabilities match exactly, and follow the 45-degree line (dotted line) downwards. The apparent calibration curve (red line) represents the calibration of the model in the development dataset, while the bias-corrected calibration curve (green line) is the calibration result after correcting for optimism using 1000 bootstrap resamples. The brier is 0.170 and the model has good calibration.

During a multivariate analysis aimed at evaluating the impact of different risk factors on the development of postoperative TND, hemopericardium, consciousness disorders, aortic valve replacement, aortic arch surgery, CPB, Post-RBP Delta, and Post-RBP Theta were identified as significant variables influencing TND. In particular, the presence of hemopericardium increased the risk of postoperative TND by 5.929 times (Table 2). The multivariable analysis conducted in this study revealed that CPB, Post-RBP Delta, and Theta were significantly associated with postoperative TND in the development cohort. In order to delve deeper into these discoveries, the TND model encompassing Post-RBP Delta and Theta was constructed, showcasing an impressive area under the receiver operating characteristic (AUROC) of 0.800 (0.730–0.871), as depicted in Fig. 3. Internal validation of the model using Bootstrap self-sampling revealed good calibration, with a brier of 0.178 (Fig. 4).

### Construction and decision curves of the nomogram for TND

**Figure 5:**
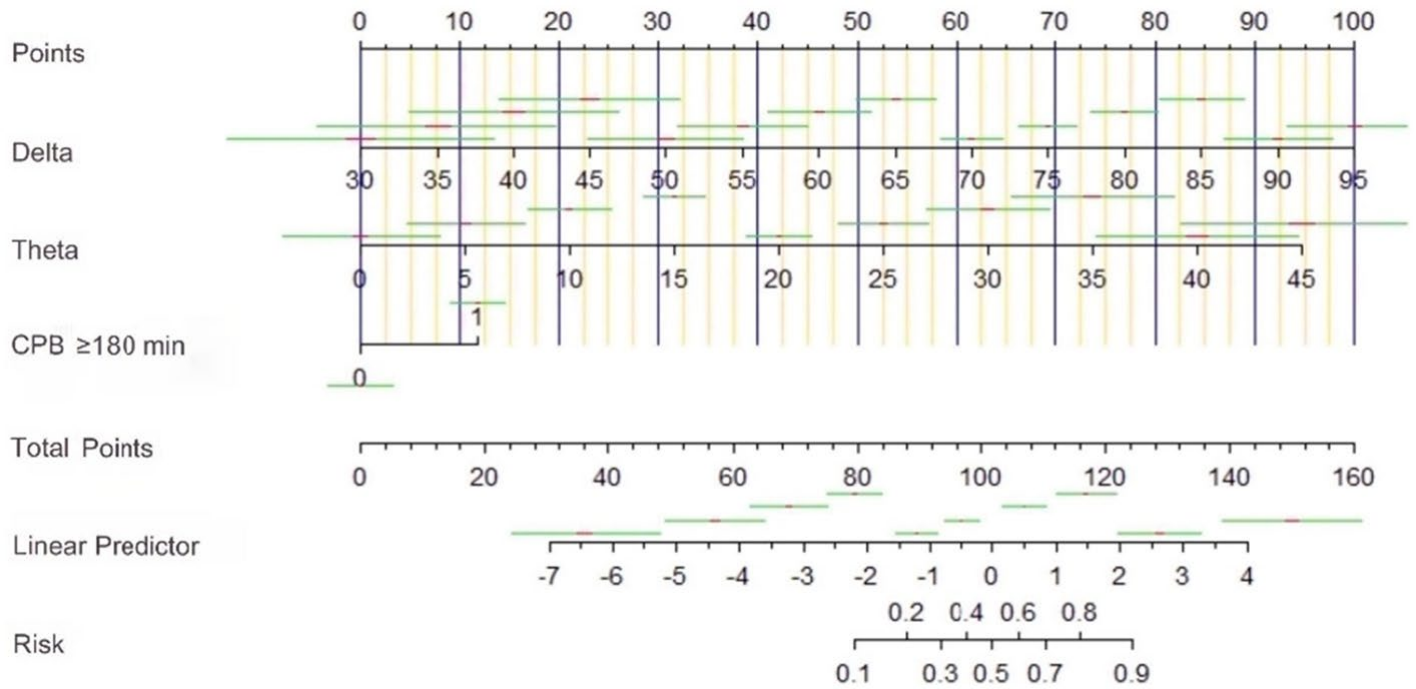
Diagnostic nomogram of Model for predicting TND after surgery for TAAD.

**Figure 6:**
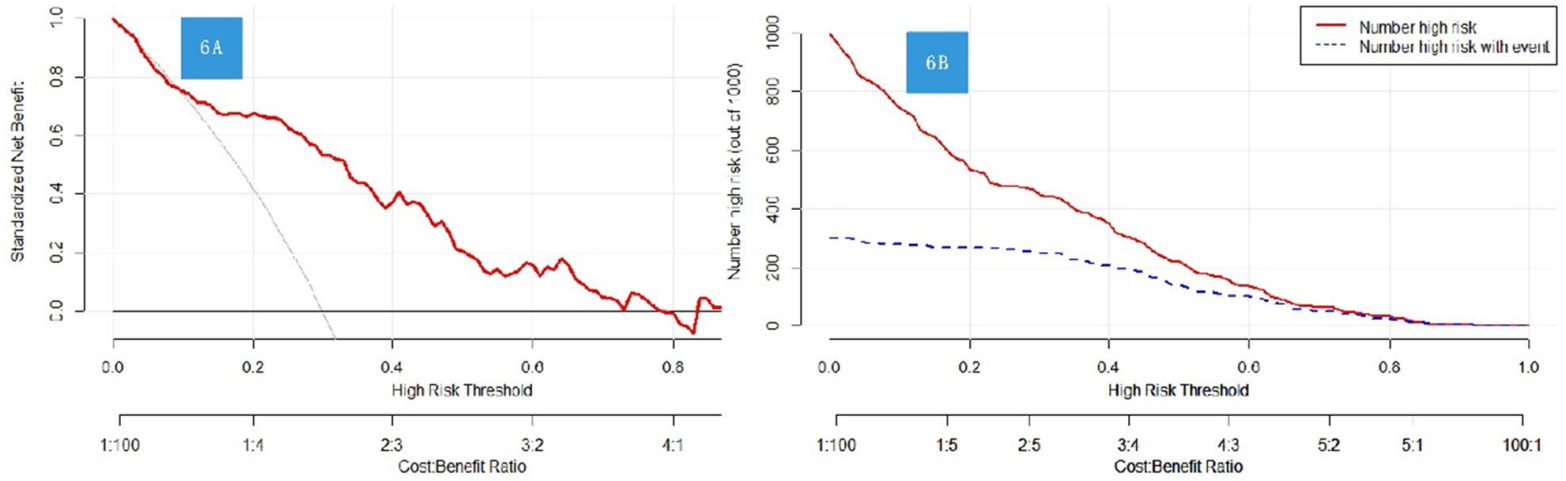
(**A**) The DCA shows the clinical net benefit of Model for TND. (**B**) The clinical impact curve of predictive Model for TND.

We developed a predictive model for postoperative TND, and created nomograms to facilitate its use in clinical practice (Fig. 5). To evaluate the clinical relevance of our models, we conducted decision curve analysis (DCA), which revealed that our models have a higher net benefit compared to default strategies only at threshold risks, rather than treating all patients or not treating any patients. Specifically, our DCA plots demonstrated that interventions based on our predictive models are most beneficial for patients with a threshold risk range of 0% to 60% for TND. Importantly, our nomograms demonstrated good clinical impact, as the predicted probabilities of TND closely aligned with actual postoperative TND, as illustrated in Fig. 6A,B.

## Discussion

Despite the utilization of cerebral protection techniques, such as hypothermia, cerebral perfusion, and blood gas management, the incidence of postoperative neurological injury following TAAD remains unchanged with the continuous advancement of surgical and monitoring technologies. Postoperative delirium and delayed awakening continue to have a high incidence rate, resulting in prolonged mechanical ventilation and hospitalization time for patients. To date, there is no effective predictive model or treatment method available for these complications.

Delayed awakening primarily refers to a prolonged recovery time following general anesthesia, though there is ongoing debate regarding its precise clinical definition. In our cardiac intensive care unit, we have been committed to promoting rapid postoperative recovery by removing endotracheal tubes within six hours after cardiac surgery. Accordingly, we define delayed awakening as failure to regain consciousness within six hours following surgery. Notably, the time window for acute stroke from onset is also six hours.

In our study, we present a novel approach for evaluating nerve function recovery in patients undergoing surgery for TAAD. Specifically, we utilized postoperative QEEG monitoring to assess the incidence of postoperative TND, which was found to be consistent with previous research findings^6,8^. Additionally, we developed a simple line graph model, based on relative power and clinical indicators, to estimate the occurrence of postoperative TND, which exhibited excellent predictive accuracy and clinical utility. Our results highlight the potential of QEEG monitoring as a valuable tool for assessing nerve function recovery in TAAD patients, and suggest that our line graph model may have practical applications in clinical settings.

Prior factors and surgical approach, along with preoperative hemopericardium indicating potential rupture of the aortic root, lead to a high incidence of postoperative TND and emergency surgery-related risks. However, the confidence interval is broad and further sample size is needed for verification. Patients who did not undergo aortic valve replacement had a lower incidence of postoperative TND. This can be attributed to the increased surgical difficulty due to aortic dissection involving the aortic valve, resulting in a higher incidence of postoperative TND. Our studies have shown that the incidence of TND in surgeries involving the aortic arch is lower with hemiarch replacement compared to other surgical approaches. This is primarily due to the simplification of the aortic arch surgery, reducing the surgical time and postoperative cerebrovascular complications. The risk factors not only increase the complexity of surgery but can also result in prolonged CPB time, further impacting postoperative neurological function. These findings are consistent with previous research results, highlighting the importance of recognizing and managing these risk factors to minimize the potential for neurological injury in patients undergoing cardiovascular surgery^3,32^.

Postoperative delirium is a common and expensive complication following cardiovascular surgery. Despite significant research on perioperative surgical factors, current delirium prediction models exhibit inadequate sensitivity and specificity. Recent studies^33,34^ have shown TND to be an acute, reversible cognitive dysfunction, with EEG studies identifying an increase in slow-wave to fast-wave ratio as a marker of brain injury, and slow-wave dominant EEG patterns predicting delirium and cognitive impairment. Our TND Model, incorporating RBP theta and delta, displays excellent diagnostic efficacy in predicting TND and has the potential to improve patient outcomes and enhance clinical decision-making for postoperative care.

In our study, we constructed a postoperative TND prediction model by monitoring the RBP 2 h post-surgery and considering whether the intraoperative CPB time exceeded 180 min. This model serves as a valuable tool for alerting clinical practitioners to manage patients more effectively. After all, the essence of dealing with diseases lies in prevention rather than awaiting the onset of conditions before intervening. Our model enables early intervention and risk reduction by identifying high-risk patients beforehand, allowing for the implementation of targeted preventive measures such as optimized anesthesia protocols and personalized pain management. This, in turn, can significantly improve patient prognosis by reducing the incidence of adverse outcomes associated with postoperative delirium, such as extended hospital stays and long-term cognitive decline. Moreover, predicting and preventing delirium optimizes healthcare resources and reduces medical costs, ultimately enhancing the quality of medical services and increasing patient and family satisfaction. For the model to be effective, it must undergo thorough validation across diverse populations and clinical settings, and be regularly updated to incorporate the latest medical insights, ensuring it can be seamlessly integrated into clinical practice to truly benefit patient care.

In conclusion, our study underscores the potential of postoperative RBP analysis in detecting early-stage TND among patients with TAAD. Our findings demonstrate the utility of RBP as a valuable adjunct for cardiac surgeons to evaluate postoperative cerebral function and facilitate timely interventions to optimize patient outcomes. These results have substantial implications for the development of personalized healthcare strategies for cardiovascular surgery patients, ultimately leading to improved clinical outcomes and enhanced patient care.

### Limitations

Despite the promising results, our study has some limitations and issues that need to be addressed. Firstly, individual differences in brain electrical activity due to age may affect RBP measurements, necessitating a larger sample size for validation of our findings. Secondly, the lack of external data validation in our study warrants the need for a larger sample size and multicenter studies to substantiate our results. These limitations highlight the need for further research to establish the full potential and generalizability of RBP as a diagnostic tool for early detection of TND in patients with TAAD.

### Supplementary Information

Supplementary Table 1.

## Data Availability

The authors will make the original data supporting the conclusions of this article available without undue reservation. This commitment underscores the authors' dedication to transparency and scientific rigor, as well as their recognition of the importance of data sharing for advancing scientific knowledge and promoting reproducibility in research. If you would like to obtain the data used in this work, please contact wangdongjingl@163.com.
