# Supplementary material for: Relative band power in assessing temporary neurological dysfunction post- type A aortic dissection surgery: a prospective study
Source: Sci Rep. 2024 Apr 3;14:7845. doi: 10.1038/s41598-024-58557-y (PMC10991486; doi:10.1038/s41598-024-58557-y)
Supplement: Supplementary file 1 — Supplementary Table 1. [file 41598_2024_58557_MOESM1_ESM.docx]

Supplementary Table 1: results of multivariable regression analysis of all variables for TND Post-TAAD Surgery

| Variables | B | SE | P value | OR | 95%CI |
| --- | --- | --- | --- | --- | --- |
| Sex | -1.42 | 0.77 | 0.07 | 0.24 | 0.05-1.05 |
| BMI | -0.07 | 0.07 | 0.35 | 0.94 | 0.81-1.07 |
| Hypertension | 1.15 | 0.62 | 0.06 | 3.17 | 0.96-11.33 |
| Age | -0.07 | 0.04 | 0.07 | 0.93 | 0.86-1.00 |
| Age≥60years | 2.02 | 1.06 | 0.06 | 7.50 | 1.00-66.25 |
| CAD | -1.21 | 1.67 | 0.47 | 0.30 | 0.01-7.93 |
| Hepatitis | -0.51 | 1.39 | 0.71 | 0.60 | 0.04-12.81 |
| Allergies | 0.80 | 1.36 | 0.56 | 2.22 | 0.13-28.36 |
| Nephropathy | -1.58 | 1.36 | 0.25 | 0.21 | 0.01-2.93 |
| MI | 16.97 | 6.52E+03 | 1.00 | 2.34E+07 | - |
| AF | 10.31 | 6.52E+03 | 1.00 | 2.99E+04 | - |
| Immune diseases | -19.26 | 2.35E+03 | 0.99 | 0.00 | - |
| Marfan syndrome | -1.62 | 3.28 | 0.62 | 0.20 | 0.00-34.98 |
| Malignancy | 0.44 | 2.46 | 0.86 | 1.56 | 0.01-181.55 |
| Smoking | 1.17 | 0.66 | 0.08 | 3.23 | 0.91-12.45 |
| Alcohol | 0.59 | 0.74 | 0.43 | 1.81 | 0.42-8.08 |
| Hemopericardium | 2.42 | 0.91 | 0.01 | 11.25 | 2.10-77.99 |
| Reoperation | -16.55 | 4.08E+03 | 1.00 | 0.00 | - |
| Mesenteric ischemia | 24.20 | 3.39E+03 | 0.99 | 3.24E+10 | - |
| Post-RBP data |  |  |  |  |  |
| Delta (%) | 0.79 | 121.10 | 0.99 | 2.21 | 0-1.30E+105 |
| Theta (%) | 0.87 | 121.10 | 0.99 | 2.38 | 0-1.41E+105 |
| Alpha (%) | 0.57 | 121.10 | 1.00 | 1.78 | 0-1.04E+105 |
| Beta (%) | 0.54 | 121.10 | 1.00 | 1.72 | 0-1.00E+105 |
| Consciousness disorders | -0.18 | 1.20 | 0.88 | 0.83 | 0.08-10.64 |
| Urgency of operation | -2.37 | 1.76 | 0.18 | 0.09 | 0.00-2.78 |
| AVR | -1.92 | 0.89 | 0.03 | 0.15 | 0.02-0.77 |
| Cerebral perfusion (u-ASCP) |  |  |  |  |  |
| RCP | 0.58 | 1.30 | 0.66 | 1.79 | 0.15-27.07 |
| Bi-ASCP | -2.53 | 1.24 | 0.04 | 0.08 | 0.01-0.76 |
| DHCA | 15.45 | 6.52E+03 | 1.00 | 5.15E+06 | - |
| Aortic procedure (Hemi-arch) |  |  |  |  |  |
| Fenestrated arch stent | -0.41 | 0.96 | 0.67 | 0.66 | 0.10-4.33 |
| Island-total arch replacement | -1.68 | 0.88 | 0.06 | 0.19 | 0.03-1.01 |
| Total arch replacement | -2.42 | 1.13 | 0.03 | 0.09 | 0.01-0.76 |
| CABG | 18.34 | 2.45E+03 | 0.99 | 9.23E+07 | - |
| ECMO | 21.27 | 6.52E+03 | 1.00 | 1.73E+09 | - |
| Lowest temperature | 1.84 | 0.94 | 0.05 | 6.26 | 1.08-45.75 |
| Operative time | 0.00 | 0.00 | 0.51 | 1.00 | 0.99-1.01 |
| CPB time | 0.01 | 0.02 | 0.79 | 1.01 | 0.97-1.04 |
| CPB≥180 min | 2.29 | 0.85 | 0.01 | 9.90 | 2.01-58.78 |
| Aortic cross-clamp time | 0.01 | 0.02 | 0.73 | 1.01 | 0.97-1.05 |
| HCA time | 0.02 | 0.03 | 0.53 | 1.02 | 0.96-1.09 |
| P/F | 0.00 | 0.00 | 0.36 | 1.00 | 1.00-1.01 |

AVR: Aortic Valve Replacement; BMI: Body mass index; CAD: coronary artery disease; MI: myocardial infarction; AF: atrial fibrillation; CABG: coronary artery bypass grafting; ECMO: extracorporeal membrane oxygenation; u-ASCP: unilateral antegrade selective cerebral perfusion; Bi-ASCP: bilateral antegrade selective cerebral perfusion; RCP: retrograde cerebral perfusion; DHCA: deep hypothermic circulatory arrest; MHCA: moderate hypothermic circulatory arrest; CPB: cardiopulmonary bypass; HCA: hypothermic circulatory arrest; P/F: PaO2/FiO2 ratio.
